# Effect of Zinc Oxide and Copper Sulfate on Antibiotic Resistance Plasmid Transfer in *Escherichia coli*

**DOI:** 10.3390/microorganisms11122880

**Published:** 2023-11-29

**Authors:** Otávio Hallal Ferreira Raro, Laurent Poirel, Patrice Nordmann

**Affiliations:** 1 Medical and Molecular Microbiology, Faculty of Science and Medicine, University of Fribourg, Chemin du Musée 18, 1700 Fribourg, Switzerland; otavio.hallalferreirararo@unifr.ch (O.H.F.R.); patrice.nordmann@unifr.ch (P.N.); 2 Swiss National Reference Center for Emerging Antibiotic Resistance (NARA), University of Fribourg, 1700 Fribourg, Switzerland; 3 Institute for Microbiology, Lausanne University Hospital and University of Lausanne, 1015 Lausanne, Switzerland

**Keywords:** *Escherichia coli*, One Health, sub-dosage, zinc oxide, copper, plasmid transfer

## Abstract

Heavy metals such as zinc (Zn) and copper (Cu) may be associated with antibiotic resistance dissemination. Our aim was to investigate whether sub-lethal dosage of Zn and Cu may enhance plasmid transfer and subsequently resistance genes dissemination. Plasmid conjugation frequencies (PCF) were performed with *Escherichia coli* strains bearing IncL-*bla*_OXA-48_, IncA/C-*bla*_CMY-2_, IncI1-*bla*_CTX-M-1_, IncF-*bla*_CTX-M-1_, and IncX3-*bla*_NDM-5_ as donors. Mating-out assays were performed with sub-dosages of zinc oxide (ZnO) and Cu sulfate (CuSO_4_). Quantification of the SOS response-associated gene expression levels and of the production of reactive oxygen species were determined. Increased PCF was observed for IncL, IncA/C, and IncX3 when treated with ZnO. PCF was only increased for IncL when treated with CuSO_4_. The ROS production presented an overall positive correlation with PCF after treatment with ZnO for IncL, IncA/C, and IncX3. For CuSO_4_ treatment, the same was observed only for IncL. No increase was observed for expression of SOS response-associated genes under CuSO_4_ treatment, and under ZnO treatment, we observed an increase in SOS response-associated genes only for IncX3. Our data showed that sub-dosages of ZnO and CuSO_4_ could significantly enhance PCF in *E. coli*, with a more marked effect observed with IncL, IncA/C, and IncX3 scaffolds. Our study suggested that use of certain heavy metals is not the panacea for avoiding use of antibiotics in order to prevent the dissemination of antibiotic resistance.

## 1. Introduction

The dissemination of antimicrobial resistance poses a significant threat to global public health concerning humans, animals, and the environment. Consequently, the World Health Organization and the World Organization for Animal Health have proposed some plans to enhance the rationale for use of antibiotics in humans and animals, aiming to control their wide administration and subsequently decrease the corresponding selective pressure [1,2].

The spread of antimicrobial resistance is highly associated with the excessive usage of antibiotics, leading to selective pressure. However, there is an increasing number of reports associating heavy metals [3,4] and other compounds such as detergents [5], nanomaterials [6], and pesticides [7] as promoters for the dissemination of antibiotic resistance. Such spread is mainly linked to plasmids carrying resistance genes in Gram negatives among isolates identified in humans [8], in the food chain [9,10,11], or in the environment [12].

Metals such as copper (Cu) and zinc (Zn) are extensively used in agricultural settings and together with cadmium (Cd) and lead (Pb) constitute the four most abundant heavy metals in the environment [13]. Once there is an escalation of livestock and agricultural production linked with an increase in the use of pesticides, fertilizers, and manure, the presence of metals is continuously accumulating in soil, water, and wastewater [13]. 

Zinc oxide (ZnO) is used as a growth promoter and to prevent or decrease the intensity of post-weaning diarrhea (PWD) in piglets and has been proposed as an alternative to antibiotics [14]. ZnO has also been associated with the food packaging industry, and it might be an alternative to improve shelf life, quality, and safety of products such as salmon without causing side effects [15]. ZnO is less toxic and showed better performance as a growth promoter when compared with other inorganic forms of Zn ions [16]. However, due to environmental concerns and the risk for an increased co-selection of antimicrobial resistant bacteria, the Committee for Medicinal Products for Veterinary Use (CVMP) re-evaluated the benefits/risks for ZnO use [17]. The CVMP concluded that the benefits of treatments of ZnO for preventing diarrhea in pigs did not compensate for the environmental risk generated with its use and decided to ban the use of ZnO beginning in June 2022 within the Europe Union (EU). However, it is still used in the rest of the world [17].

Cu (heptanoate, chloride, sulphate, oxide, gluconate, and other forms) is also used for promoting animal growth and has been used as an additive in animal feed for food-producing animals [18]. Moreover, it has been used in plants as a fungicide and to control foot rot in cattle and sheep, an infectious disease of the subcutaneous tissue and interdigital skin [18,19]. However, its excessive intake has been associated with liver damage in humans [20] and environmental pollution [13]. Of particular concern is the demonstration that Cu nanoparticles and Cu ions may increase plasmid conjugation frequency (PCF) [3].

*Escherichia coli* may act an opportunistic pathogen capable of donating resistance genetic elements to other pathogenic *E. coli* or to different bacteria belonging to other species, particularly Enterobacterales [21,22]. It causes urinary and gastrointestinal tract infections, bloodstream infections, meningitis, and septicemia in humans [23] and is also a significant pathogen causative of diarrhea in animals [24].

Plasmids encoding antibiotic resistance in Enterobacterales belong to many different incompatibility groups, including IncA/C, IncI1, IncF, and IncX3 types that have been identified either from human or animal sources [25,26,27,28,29,30]. IncL plasmids are more often associated with human *E. coli* isolates but are increasingly reported from companion animals [31,32] and in the food chain [33,34,35]. Some of these plasmids are responsible for the global spread of clinically relevant Ambler class A extended-spectrum ß-lactamases (ESBLs) (e.g., CTX-Ms), class B (NDM-, IMP-, VIM-like), class C (AmpCs; e.g., CMY-like), or class D (e.g., OXA-48) ß-lactamases [25,26]. 

Stress conditions such as starvation, temperature changes, and exposure to antibiotics and heavy metals may play an important role in potentially inducing acquisition of antibiotic resistance genes through horizontal gene transfer (HGT) [3,4,36,37]. In *E. coli*, these stress conditions increase the production of reactive oxygen species (ROS) levels, causing harmful effects through DNA, protein, and lipid damage alterations, inducing and activating the SOS response [38]. The stress-associated SOS response is an organized cellular response to DNA damage and replication blockage. SOS system key protein RecA (encoded by *recA* gene) is crucial to restore DNA and bacterial subsistence [39]. Altogether, RecA and more than 40 proteins compose the SOS regulon in *E. coli*, being involved in DNA repair, recombination, translational synthesis, biofilm formation, and HGT [40]. Thus, stress conditions can induce mutagenesis and HGT through the SOS response, eventually enhancing the dissemination of resistance genes [41]. It has been shown that presence of zinc oxide can lead to a decreased virulence in *E. coli* by inducing stress response and inhibiting the SOS response [42]. Some studies have demonstrated that Zn oxide may protect LexA to be cleaved (early step of the SOS response pathway) by interfering with RecA [42]. Thus, it might be speculated that the presence of Zn, by preventing the triggering of the SOS response, might result in a decrease of the antibiotic resistance dissemination.

There is not a consensus on the real effect that heavy metals may have on conjugation efficiency in *E. coli*. For instance, Palm et al. [43] showed that sodium arsenite, zinc chloride, and cooper sulfate decreased conjugation frequency in incF-plasmid in *E. coli*; however, other studies showed that Zn nitrate and CuSO_4_ increased conjugation frequency for *E. coli* and *Salmonella enterica* depending on three different factors, the plasmid scaffold involved, the strain, or the concentration of heavy metals used [3,44].

In order to better understand the effect that heavy metals have on conjugation frequency, and considering that resistance determinants can be exchanged between environment, animal, and human bacterial communities, our aim was to investigate whether sub-lethal dosage of widely used metals (Zn and Cu) in veterinary and agricultural settings could enhance PCF and consequently resistance genes’ dissemination.

## 2. Materials and Methods

### 2.1. Bacterial Strains and Plasmids 

Five *E. coli* strains were used as plasmid donors in our conjugation experiments: (i) *E. coli* harboring an IncL plasmid (N502), which carries the *bla*_OXA-48_ carbapenemase gene [45]; (ii) *E. coli* DH10B harboring an IncA/C plasmid (R2672) carrying the *bla*_CMY-2_ gene encoding resistance to expanded-spectrum cephalosporins (this study); (iii) *E. coli* harboring an IncI1 (R975) plasmid carrying the ESBL gene *bla*_CTX-M-1_ [11]; (iv) *E. coli* harboring an IncF plasmid (R5059) also carrying *bla*_CTX-M-1_ [46]; (v) and *E. coli* harboring an IncX3 plasmid (R5998) carrying the carbapenemase gene *bla*_NDM-5_ [47]. Those plasmids and genes were chosen in order to mirror those frequently identified as sources of antibiotic resistance of concern for human medicine. The nalidixic-acid resistant *E. coli* strain JM109 was used as the recipient for conjugation experiments [48]. 

### 2.2. Heavy Metals and Antimicrobial Susceptibility Testing

ZnO (Sigma-Aldrich, Saint Louis, MO, USA) and CuSO_4_ (copper (II) sulphate pentahydrate; Sigma-Aldrich), metals widely used in food-producing animal farms and agricultural settings were selected for this study. The microdilution method was performed in Mueller-Hinton medium (MH) (Bio-Rad, Cressier, Switzerland) to determine the minimum inhibitory concentration (MIC) according to the Veterinary Antimicrobial Susceptibility Testing (VetCAST) and Clinical and Laboratory Standards Institute Bacteria Isolated from Animals (CLSI VET) recommendations and guidelines [49,50].

### 2.3. Mating-Out Assays

Conjugation assays were performed as described [51], with some modifications. Briefly, (i) plasmid donors and recipients were pre-inoculated in Luria-Bertani medium (LB) (Bio-Rad) overnight at 37 °C with shaking; (ii) then, donors (with or without sub-lethal concentrations of heavy metals) and recipients were once more incubated in LB at 37 °C with shaking for 5 h. (iii) Cells were centrifuged at 3000× *g* for 10 min and resuspended in the residual culture medium (3 mL) after pouring the supernatant. (iv) Donors and recipients were respectively mixed in a 1:4 volume ratio and centrifuged at 3000× *g* for 10 min before being transferred on filters (0.22 µm; Merck Millipore, Burlington, MA, USA) and incubated in LB plates (Carl Roth, Karlsruhe, Germany) at 37 °C without shaking for 4 h. (v) Cells were washed away from the filters using sodium chloride (NaCl 0.85%—Sigma-Aldrich), and the mixture was vortexed to stop conjugation. (vi) Serial dilution was performed using NaCl, and the mixture was plated on LB agar plates containing 50 mg/L of ampicillin (Sigma-Aldrich) or temocillin (Eumedica SA, Brussels, Belgium) (for quantifying donors and transconjugants) and 50 mg/L of ampicillin or temocillin (selection of the OXA-48 resistance marker) plus 50 mg/L of nalidixic acid (Sigma-Aldrich) (for quantifying transconjugants only) and finally incubated overnight at 37 °C. Conjugation frequencies (CF) were calculated by dividing the number of transconjugants by the number of donors [51].

### 2.4. Fluorescent Reactive Oxygen Species (ROS) Detection 

ROS detection was performed to evaluate whether *E. coli* cells would indeed be under stress and therefore reacting by increasing their ROS production in response to exposure to sub-inhibitory concentrations of heavy metals. Donor strains were submitted to treatment with and without metals and incubated at 37 °C for 5 h with shaking, as described above. After that, the ROS detection assay was done as described by Castro-Alférez et al. [52], with adjustments. Measurements of chemical hydrolysis of the probe 2,7-dichlorodihydrofluorescein diacetate (DCFH-DA; Sigma-Aldrich) in the fluorescent compound 2,7-dichlorofluorescein (DCF) were detected by fluorescence spectroscopy at 522- and 498-nm emission and excitation wavelengths, respectively. Briefly, cells were centrifuged at 5000× *g* for 10 min, and the supernatant was poured off. After that, cells were washed with phosphate-buffered saline (PBS, 0.01 mM—Sigma-Aldrich) and centrifuged at 5000× *g*. This step was repeated twice to avoid any interaction between the fluorescent probe and the medium/metals used in the former steps. DCFH-DA 10 µM was added for a 500 µL final volume and incubated at 37 °C for 20 min in a dark setting. Fluorescence detection was performed in 96-well solid bottom black plates using the TECAN 200Pro (Tecan, Switzerland) fluorimeter. Hydrogen peroxide 2.5 mM (H_2_O_2_—Sigma-Aldrich) was used as a positive control for the experiment.

### 2.5. mRNA Extraction and cDNA Synthesis

The total RNA was extracted using the Quick-RNA^TM^ MiniPrep kit (Zymo Research, Irvine, CA, USA) after the strains were submitted to the initial treatment with or without metals and incubated at 37 °C for 5 h with shaking. Then, to remove contaminating DNA from RNA preparations and DNase and divalent cations from the samples, a turbo DNA-*free*^TM^ kit (Invitrogen, Waltham, MA, USA) was used. cDNA synthesis was performed with the LunaScript^®^ RT SuperMix kit (New England BioLabs, Ipswich, MA, USA). All experiments were performed following the manufacturer’s instructions. Finally, cDNA samples were measured to assure purity and standard concentration quantification using the NanoDrop 2000 spectrophotometer (Thermo Scientific, Reinach, Switzerland). 

### 2.6. RT-qPCR 

Quantitative real-time PCR was done using the Rotor-Gene Q cycler (Qiagen, Hilden, Germany). The primers used in this experiment were the 16S rRNA-encoding gene (reference), the *recA* gene (recombinase), and the gene *sfiA* (cell division inhibitory) [53]. Reactions were established with a total volume of 20 µL using a GoTaq^®^ qPCR Master Mix kit (Promega, Fitchburg, WI, USA). The cycle threshold (*C_T_*) values were analyzed by the 2^−ΔΔCT^ method [54]. Relative expression levels were calculated by comparing the results with the control samples, and the condition values were corrected with the appropriate reference gene.

### 2.7. Statistical Analyses

The tests were done in three independent replicates. Data were analyzed by unpaired *t*-test with a two-tailed *p*-value analyses using GraphPad Prism Software version 9.3.1. Statistically significant results were defined with a confidence level of 95% (*p* < 0.05).

## 3. Results

### 3.1. Increased PCF in Presence of Low Concentrations of Heavy Metals 

ZnO and CuSO_4_ were tested at sub-inhibitory concentrations to evaluate their ability to influence PCF. Plasmids of IncL, IncA/C, IncI1, IncF, and IncX3 types harbored by distinct *E. coli* isolates were respectively used as templates for conjugation assays, using *E. coli* JM109 as the recipient strain. The different *E. coli* donors were incubated in the presence of concentrations corresponding to half of the MICs for ZnO and CuSO_4_ (therefore distinct for each donor strain).

Conjugation experiments showed significant increases on PCF for plasmids of type IncL, IncA/C, and IncX3 when treated with ZnO. The fold changes observed in IncL, IncA/C, and IncX3 were respectively 13.8- (*p* < 0.01), 9.9- (*p* < 0.05), and 18.4-fold (*p* < 0.05) (Table 1). By contrast, no significant increase of PCF was observed for *E. coli* carrying IncI1 or IncF plasmids when treated with ZnO.

When *E. coli* cultures were incubated with sub-inhibitory concentrations of CuSO_4_, a significant impact on the PCF was observed only with the IncL-type plasmid, with a 17-fold increase (*p* < 0.05) (Table 2). By contrast, no impact on PCF was observed for IncA/C-, IncI1-, IncF-, and IncX3-type plasmids. Those data were achieved by comparing with the control experiment performed in the absence of metals for each strain.

### 3.2. Sub-Inhibitory Concentrations of ZnO and CuSO_4_ Enhance Oxidative Stress Response in E. coli 

**Figure 1:**
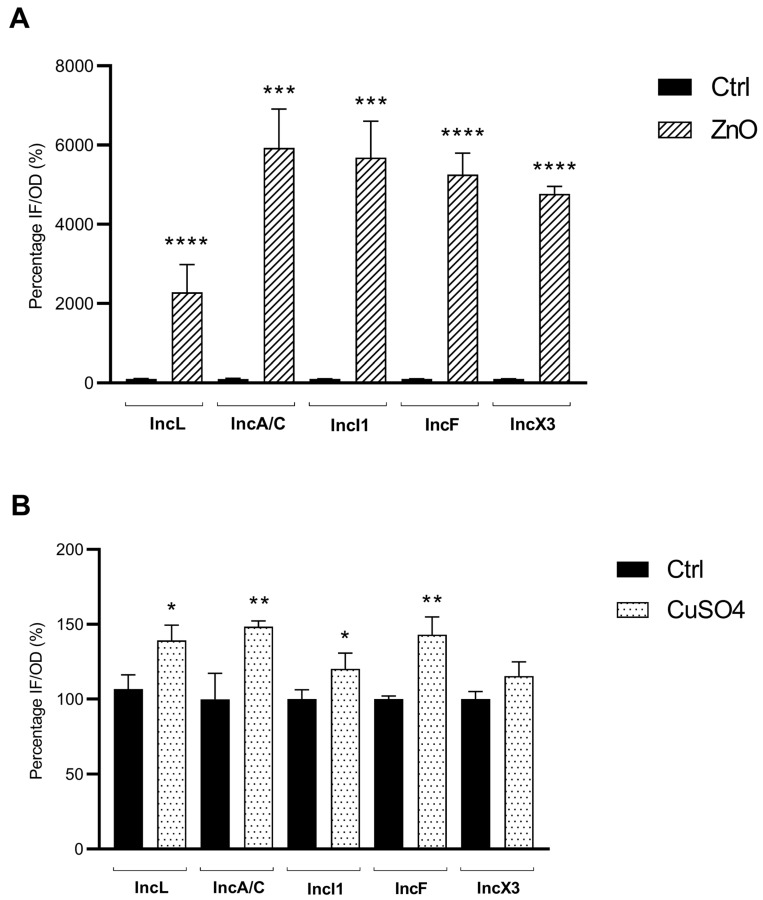
Reactive species of oxygen (ROS) experiment by fluorimetry. (**A**) ROS production for five *E. coli* isolates carrying distinct plasmids after treated with sub-inhibitory concentration of zinc oxide (ZnO); (**B**) ROS production for the *E. coli* isolates carrying distinct plasmids after treated with sub inhibitory concentration of copper sulphate (CuSO_4_). IF/OD, intensity of fluorescence/optical density; CTRL, control (no treated); ZnO (IncL and IncI1, 512 µg/mL; IncA/C, IncF, and IncX3, 128 µg/mL); CuSO_4_ (all plasmids, 512 µg/mL). Data are presented in means and standard deviations. *, *p* < 0.05 (*, against the respective control); **, *p* ≤ 0.01; ***, *p* ≤ 0.001; ****, *p* ≤ 0.0001.

Measurements of the ROS production were performed for all *E. coli* strains in the presence or absence (control) of sub-inhibitory concentrations of ZnO and CuSO_4_. After treatment with ZnO, an overall positive correlation between ROS production and increase in PCF was observed. Strains harboring plasmids IncL, IncA/C, and IncX3 showed a significant increase of 22.8- (*p* ≤ 0.0001), 59.3- (*p* ≤ 0.001), and 47.7-fold (*p* ≤ 0.0001) of the ROS production when compared with their respective controls (Figure 1A). Conversely, for strains R975-IncI1 and R5059-IncF, significant increases in ROS production of respectively 56.9- (*p* ≤ 0.001) and 52.6-fold (*p* ≤ 0.0001) were observed, which did not correlate with PCF after ZnO treatment (Figure 1A and Table 1). 

Upon treatment with CuSO_4_, a positive correlation was found between the increased production of ROS and the increased PCF observed with only a single isolate, namely N502-IncL, the increase in ROS production being slight but significant (1.3-fold [*p* < 0.05]) when compared with the control (Figure 1B). A different effect was observed for isolates harboring IncA/C, IncI1, and IncF-type plasmids, for which a significant increase in ROS production was observed (respectively 1.5- [*p* ≤ 0.01], 1.2- [*p* < 0.05], and 1.4-fold [*p* ≤ 0.01]), but with no PCF induction being observed (Figure 1B and Table 2). Results for the strain carrying the IncX3-type plasmid showed a positive correlation between the techniques; neither a significant increase in ROS production (*p* = 0.0662) nor an increase in PCF were observed.

### 3.3. Heavy Metals Sub-Lethal Dosage Treatment Does Not Alter SOS-Responsive Gene Expression for the Majority of the E. coli Strains

The expression levels of the *recA* and *sfiA* genes was evaluated for the different *E. coli* strains with and without treatment under sub-inhibitory concentrations of ZnO and CuSO_4_ in order to evaluate whether the recombinase gene *recA* (key factor for triggering the SOS response) and the cell division inhibitory encoding gene *sfiA* would be affected upon presence of heavy metals sub-inhibitory concentrations. 

For strains N502-IncL, R2672-IncA/C, R975-IncI1, and R5059-IncF, treatment with sub-inhibitory concentrations of ZnO resulted in no significant increase in *recA* and *sfiA* gene expression levels (Table 3). Similar results were observed when treating the same strains with sub-inhibitory concentrations of CuSO_4_. However, for strain R5998-IncX3, although no increase in the expression level of those SOS response-associated genes was observed when treated with CuSO_4_, increases of 38.1- (*p* < 0.01) and 45.9-fold (*p* < 0.01) of the *recA* and *sfiA* respective gene expression levels were surprisingly observed after treatment with sub-inhibitory concentration of ZnO.

## 4. Discussion

In accordance with the One Health concept, it is now admitted that the problems of antimicrobial resistance encountered in humans, animals, and environment bacterial communities are linked. Bacteria are indeed continuously exchanging genetic determinants, thus contributing to the public health concern towards antibiotic resistance dissemination [8]. *E. coli* is the best example of bacterial species that may promote the transfer of resistance genes to humans [55], to food-producing [9,10,11] and companion animals [56], and within the environment [12]. Although antibiotics present in sub-inhibitory concentrations may contribute to the development of antibiotic resistant strains, they have also been shown to participate in the dissemination of the corresponding resistance genes. Indeed, for those antibiotic resistance genes being plasmid-borne, one of the factors leading to such enhanced dissemination is the increase of PCF. Besides the role of antibiotics, there is also a discussion regarding the potential impact of heavy metals in this dissemination process [3,4]. Such questioning makes sense when considering that use of some heavy-metal compounds could be considered as an alternative to antibiotic usage for preventing bacterial infections. This is, for instance, the case for ZnO when it is used as a prophylactic agent for preventing diarrhea in pig production [14,57].

The antibacterial action of ZnO is not completely elucidated. ZnO has attributes of a semiconductor and transition metal, and its binding energy allows for a substantial oxidative character, which can generate reactive oxygen species, promoting bacterial cell wall damage [58,59,60]. This metal can also release its ionic form (Zn^2+^), provoking cell membrane disruption [61]. Moreover, ZnO can also inhibit bacterial adhesion to cells [62]. Here we showed that ZnO had a significant induction effect in the PCF for *E. coli* strains harboring a series of distinct plasmids encoding antibiotic resistance. It was previously shown that Zn or ZnO could increase plasmid mobilization capacity for IncI1 plasmids [63,64] in *E. coli*, suggesting that the stress that the addition of Zn caused to the bacteria plays a role in an increased transmission of plasmids from cells to cells [65]. Another study evaluated the effect of Zn nitrate on PCF by using *E. coli* and *Salmonella enterica* as donor and recipient, respectively [44], and showed that Zn exposure influenced conjugation frequencies in only one out of three recipient strains tested, suggesting a concentration- or strain-dependent effect [44]. Likewise, we also observed that sub-inhibitory concentrations of Zn had no effect on PCF for two out of the five *E. coli* strains possessing different plasmid scaffold tested, suggesting that this phenomenon could be strain- but possibly also plasmid scaffold-dependent.

The mode of action of Cu is also not totally understood, but it has been attributed to the release of the Cu ions Cu^+^ and Cu^+2^ that irreversibly damage bacterial cell membranes, inducing generation of ROS [66]. Our results showed an increased PCF enhanced by CuSO_4_ for only one of the strains tested. In a previous study, Wang et al. [67] showed the effect of typical heavy metals on an RP4 plasmid (IncP) in freshwater microcosms, demonstrating that CuSO_4_, Zn sulfate (ZnSO_4_), and lead sulfate (PbSO_4_) promoted conjugative transfer of RP4. Similar to our observations with the IncL-type plasmid, they showed that CuSO_4_ had the ability to significantly increase the conjugation frequency. Similarly, Zhang et al. [3] also showed that exposing bacteria to Cu nanoparticles or Cu^+2^ at sub-inhibitory concentrations significantly enhanced PCF across genera (with *E. coli* as donor and *Pseudomonas putida* as recipient). Of note, instead of dividing the number of transconjugants by the number of donors, Zhang et al. and Wang et al. calculated PCF by normalizing the total colonies of transconjugants to the total colonies of recipients [3,67]. However, Buberg et al. [68] obtained opposite results, showing a reduction in PCF and expression of conjugation genes (*traB* and *nikB*) after exposure to Zn chloride (concentration ranging from 0.03 µg/mL to 3 mg/mL) and CuSO_4_ (from 0.01 µg/mL to 1 mg/mL) for ESBL-producing *E. coli*. These data corroborate with our results observed for N502-IncL isolate when treated with low dosage of CuSO_4_ and with both R975-IncI1 and R5059-IncF when treated with ZnO. This further reinforces the hypothesis that plasmid transfer induction by heavy metals might be strain- or plasmid-dependent. 

We identified here a significant increase in ROS production for all isolates upon treatment with sub-inhibitory concentrations of ZnO and for all but one isolate upon treatment with CuSO_4_. Despite significant increases in PCF and ROS production being observed for strains R2672-IncA/C and R5998-IncX3 upon treatment with ZnO and for N502-IncL upon treatment with both ZnO and CuSO_4_, this pattern was not observed for either R975-IncI1 and R5059-IncF when treated with ZnO or CuSO_4_ or for R2672-IncA/C and R5998-IncX3 when treated with CuSO_4_. Considering that ROS induced by metals generates oxidative stress in *E. coli*, leading to the inhibition of protein synthesis and DNA replication, we can therefore speculate that ZnO and CuSO_4_ may be toxic, inhibiting crucial energetic machinery determinants, including *tra* gene encoding for proteins involved in the conjugation process [69] in those isolates for which no association was observed. 

ZnO halts SOS response in *E. coli* by blocking *recA* expression [47,48]. Bunnel et al. [42] demonstrated the inhibition of the *recA* gene expression in those conditions, leading to a protection of the LexA repressor from RecA-mediated cleavage, which is a critical and early step for initiating the SOS response [70]. This could explain why no significant increase in *recA* expression levels was observed in our experiments after treatment with ZnO. Similarly, it might explain the lack of increased expression of the *sfiA* gene encoding the SfiA protein, an SOS-associated division inhibitor inhibiting the FtsZ ring formation, being an essential step for cell division regulation during the SOS response [71]. Additionally, some conjugative plasmids are known to express anti-SOS response genes (for example *psiB* gene) to quench the activation of the SOS response upon entry into a new host [72]. The lack of increased expression of the *recA* and *sfiA* genes after treatment with ZnO observed for several strains might also be explained by the insufficient expression levels of the anti-SOS genes, hence a lack of significant alleviation of the SOS response, as showed for IncF plasmids in previous studies [72,73].

Although an increased PCF was identified in our experiments after treating N502-IncL, R2672-IncA/C, and R5998-IncX3 strains with metals, it remains unclear why this effect was not observed for the two other strains, R975-IncI1 and R5059-IncF. Further investigations will therefore be needed to try to find some explanations at the molecular level.

Sub-inhibitory concentrations are particularly important to take into account in veterinary practice since the delivery of antibiotics or other compounds like heavy metals are frequently associated with inconsistencies in animal husbandries. One such example is food supplementation, which essentially results in variable concentrations of these compounds while eating. Furthermore, the environment can be contaminated with residual concentrations of heavy metals.

As a conclusion, our data showed that sub-inhibitory dosages of ZnO and CuSO_4_, which are widely used heavy metals in veterinary and agricultural settings, could significantly enhance PCF in *E. coli*, with a more marked effect being observed for plasmids possessing IncL (ZnO and CuSO_4_), IncA/C (ZnO), and IncX3 (ZnO) scaffolds. Our study suggested that use of heavy metals is not the panacea for avoiding the use of antibiotics in order to prevent the dissemination of antibiotic resistance. 

## Figures and Tables

**Table 1:** Conjugation frequency and fold change in filter mating assays for *E. coli* isolates carrying different incompatibility family plasmids submitted to sub dosage of zinc oxide.

		Control		½ MIC	
Plasmids	MIC (µg/mL)	CF	FC	CF	FC
IncL	1024	1.19 × 10^0^ ± 2.65 × 10^−2^	1	1.38 × 10^1^ ± 4.54 × 10^0^	13.8 **
IncA/C	256	1.00 × 10^0^ ± 5.30 × 10^−1^	1	9.93 × 10^0^ ± 4.54 × 10^0^	9.9 *
IncI1	1024	9.99 × 10^−1^ ± 7.98 × 10^−1^	1	3.77 × 10^0^ ± 2.19 × 10^0^	3.8
IncF	256	6.20 × 10^−1^ ± 1.07 × 10^0^	1	1.54 × 10^−1^ ± 1.44 × 10^−1^	0.12
IncX3	256	9.99 × 10^−1^ ± 3.83 × 10^−1^	1	1.84 × 10^1^ ± 1.03 × 10^1^	18.4 *

CF: conjugate on frequency; FC: fold change; * Bold: *p* < 0.05; ** Bold: *p* < 0.01.

**Table 2:** Conjugation frequency and fold change in filter mating assays for *E. coli* isolates carrying different incompatibility family plasmids submitted to sub dosage of copper sulphate.

		Control		½ MIC	
Plasmids	MIC (µg/mL)	CF	FC	CF	FC
IncL	1.024	1.00 × 10^0^ ± 6.78 × 10^−1^	1	1.69 × 10^1^ ± 9.86 × 10^0^	17.0 *
IncA/C	1.024	1.00 × 10^0^ ± 1.28 × 10^0^	1	1.32 × 10^0^ ± 1.40 × 10^0^	1.3
IncI1	1.024	1.00 × 10^0^ ± 1.12 × 10^0^	1	1.28 × 10^0^ ± 6.78 × 10^−1^	1.3
IncF	1.024	9.99 × 10^−1^ ± 2.27 × 10^−1^	1	1.31 × 10^0^ ± 1.12 × 10^0^	1.3
IncX3	1.024	9.99 × 10^−1^ ± 3.83 × 10^−1^	1	5.12 × 10^−1^ ± 4.49 × 10^−1^	0.5

CF: conjugation frequency; FC: fold change; * Bold: *p* < 0.05.

**Table 3:** Expression of chromosomal genes related to SOS response in *E. coli* when submitted to sub-inhibitory concentration of metals zinc oxide and copper sulphate.

		2^−ΔΔCT^		
Gene	Isolate	C	Zinc Oxide	Copper Sulphate
*recA*	N502-IncL	1	0.10 ± 0.04	2.31 ± 2.09
	R2672-IncA/C	1	3.49 ± 1.96	0.75 ± 0.10
	R975-IncI1	1	7.53 ± 5.15	0.64 ± 0.26
	R5059-IncF	1	29.08 ± 20.46	0.50 ± 0.25
	R5998-IncX3	1	38.12 ± 8.73 **	0.37 ± 0.10
*sfiA*	N502-IncL	1	0.03 ± 0.01	0.54 ± 0.44
	R2672-IncA/C	1	27.41 ± 30.75	0.40 ± 0.09
	R975-IncI1	1	26.45 ± 17.95	0.20 ± 0.06
	R5059-IncF	1	6.05 ± 3.75	0.14 ± 0.08
	R5998-IncX3	1	45.92 ± 13.40 **	1.27 ± 0.98

2^−ΔΔCT^: 2(-Delta Delta C(T)) method; C: control; ** Bold: *p* < 0.01.

## Data Availability

The datasets generated for this study are available on request to the corresponding author.
